# Longitudinal Body Composition Changes Detected by [^18^F]FDG PET/CT during and after Chemotherapy and Their Prognostic Role in Elderly Hodgkin Lymphoma

**DOI:** 10.3390/cancers14205147

**Published:** 2022-10-20

**Authors:** Domenico Albano, Francesco Dondi, Giorgio Treglia, Alessandra Tucci, Marco Ravanelli, Davide Farina, Francesco Bertagna

**Affiliations:** 1 Nuclear Medicine Department, ASST Spedali Civili Brescia, 25128 Brescia, Italy; 2 Nuclear Medicine, Department of Medical and Surgical Specialties, Radiological Sciences, and Public Health, University of Brescia, 25121 Brescia, Italy; 3 Clinic of Nuclear Medicine, Imaging Institute of Southern Switzerland, Ente Ospedaliero Cantonale, 6500 Bellinzona, Switzerland; 4 Department of Nuclear Medicine and Molecular Imaging, Lausanne University Hospital, University of Lausanne, 1015 Lausanne, Switzerland; 5 Faculty of Biomedical Sciences, Università della Svizzera Italiana, 6900 Lugano, Switzerland; 6 Division of Hematology, ASST Spedali Civili Brescia, 25128 Brescia, Italy; 7 Department of Radiology, University of Brescia and ASST Spedali Civili Brescia, 25128 Brescia, Italy

**Keywords:** PET/CT, computed tomography, lymphoma, Hodgkin, FDG, sarcopenia, body composition

## Abstract

**Simple Summary:**

The effective role of sarcopenia and body changes during treatment in elderly (>65 years) Hodgkin lymphoma (HL) is unclear. The aim of our study was to analyze the longitudinal body changes in terms of muscle and adipose area measurement using the computed tomography (CT) of positron emission tomography (PET)/CT during and at the end of chemotherapy. Moreover, we examined the prognostic role of these parameters together with the main clinical and metabolic variables. The incidence of sarcopenia increased in elderly HL during the chemotherapy course, with, e.g., the gain of adipose tissue areas. [^18^F]FDG PET/CT features (metabolic response at interim and end-of-treatment), baseline sarcopenia, and body composition changes during chemotherapy may have a prognostic role, especially for overall survival.

**Abstract:**

The aim of this retrospective study was to investigate the longitudinal body changes in terms of muscle and adipose areas and their prognostic role in elderly (>65 years) patients affected by Hodgkin lymphoma (HL). Skeletal muscle area (SMA), skeletal muscle index (SMI), visceral adipose tissue (VAT), subcutaneous adipose tissue (SAT), intramuscular adipose tissue (IMAT), and total dispose tissue (TAT) were measured using the computed tomography (CT) of fluorine-18-fluorodeoxyglucose positron emission tomography/CT ([18F]FDG PET/CT) in 88 patients who undertook baseline, interim (after two cycles of chemotherapy), and end-of-treatment (after 6 cycles of chemotherapy) PET/CT scans. Metabolic tumor volume (MTV) and total lesion glycolysis (TLG) were measured at pre-treatment PET/CT. Metabolic response applying Deauville score was evaluated at interim and end-of-treatment PET/CT. Survival curves, such as progression free survival (PFS) and overall survival (OS), were calculated for the whole population. Fifty-eight (66%) patients had sarcopenia at baseline and sarcopenia rate increased at interim scan with 68 (77%) cases and at end-of-treatment scan with 73 (83%) cases. Muscular areas (SMA and SMI) declined significantly during the treatment (*p* < 0.001), decreasing from baseline by 5% and 7% at interim and end-of-treatment evaluation, respectively. Instead, VAT, SAT, IMAT, and TAT increased significantly over this time (*p* < 0.001). Sarcopenia was significantly related with comprehensive geriatric assessment. PET/CT response at interim and end-of-treatment, MTV, TLG, and baseline sarcopenia were independent prognostic factors for PFS. Instead, metabolic response at interim and end-of-treatment PET, baseline sarcopenia, ΔSMI at interim, and ΔSMI at end-of-treatment for OS were independent prognostic factors.

## 1. Introduction

Hodgkin lymphoma (HL) is a disease that typically affects young adults, but it has a bi-modal age distribution curve, with approximately 25% of cases diagnosed in patients more than 60 years old [1], and this rate will increase in the near future considering the expected gain in median life in Western countries. The prognosis of elderly HL patients is much worse than that of younger HL patients, due to the difficulties to digest aggressive chemotherapy protocols and comorbidities [2,3]. 2-deoxy-2-[18F]fluoro-D-glucose positron emission tomography/computed tomography ([^18^F]FDG PET/CT) is a non-invasive tool already studied in elderly HL [4,5]. Particularly, Deauville scores measured at interim and end-of-treatment PET and several semiquantitative baseline PET/CT parameters, such as metabolic tumor value (MTV) and total lesion glycolysis (TLG), are significantly related to survival [4,5]. Besides these variables, sarcopenia also seems to have a prognostic impact for lymphoma [6]. Sarcopenia is considered a syndrome characterized by a progressive and diffuse loss of skeletal muscle mass and strength with a high risk of adverse events, e.g., physical disability, poor quality of life, and even exitus [7,8]. Nowadays, a large number of examinations are available for the definition of sarcopenia in clinical practice and in research fields. Now, CT is considered the best tool for the non-invasive assessment of muscle and fat quantity/mass. To define the muscular mass, conventionally, we can measure a cross-sectional area of specific muscle groups located at the third lumbar vertebra called skeletal muscle area (SMA), after adjusting for stature defined as skeletal muscle index (SMI). There are also preliminary data concerning the possibility to measure muscle and fat areas with the low-dose CT component of PET/CT scan with high accuracy and reproducibility [9]. However, a parameter not well evaluated is the evolution of body composition during treatment in oncological patients. Initial studies reported body composition changes occurring after therapy in lymphoma, such as hematopoietic cell transplantation [10,11,12] or systemic therapy (chemotherapy) [13], but with controversial results. Thus, the first aim of this retrospective study was to describe the longitudinal body changes in terms of muscle and adipose areas measured by baseline, interim, and end of treatment [^18^F]FDG PET/CT in elderly patients affected by HL undergoing chemotherapy. The other aim was to investigate the prognostic impact of the body composition changes.

## 2. Materials and Methods

**Figure 1:**
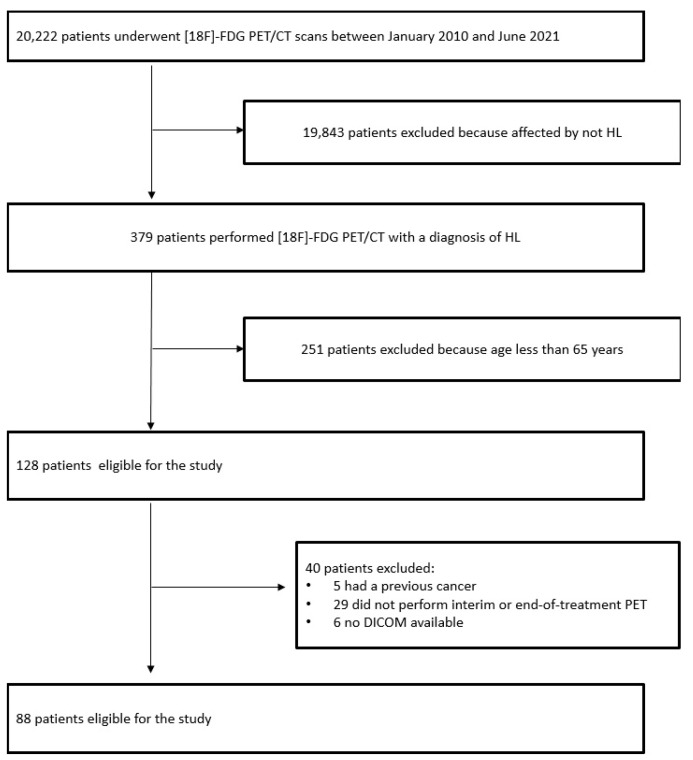
Selection scheme of the patients eligible for the study.

Between January 2010 and June 2021, we retrospectively reviewed about 42,265 [^18^F]FDG PET/CT scans performed on 20,222 patients in our Nuclear Medicine Center. Among them, all patients with a confirmed histological diagnosis of HL were included. To be eligible for this study, inclusion criteria were: (a) aged ≥ 65 at the time of diagnosis; (b) availability of [^18^F]FDG PET/CT scan at baseline (before any kind of treatment), after 2 cycles of chemotherapy (interim PET), and after 6 cycles of chemotherapy (end-of-treatment PET); (c) availability of digital DICOM files for the measurements of muscular and adipose areas; (d) no previous other oncological diseases; (e) at least 12 months of follow-up. Finally, data from 88 patients were collected (Figure 1). The main medical data of our patients were reviewed focus on several epidemiological (age at diagnosis, gender, weight, height, BMI), clinical (histological variant, stage, B symptoms, bulky disease, lactate-dehydrogenase (LDH) level), sarcopenic, and functional characteristics by [^18^F]FDG PET/CT, therapy modality, and follow-up information. These patients received therapies according to the institution’s standard protocol with chemotherapy regimen. Sixty-three patients were treated according to ABVD (Doxorubicin, Bleomycin, Vinblastine, Dacarbazine) or ABVD-like regimen, while the remaining 25 with other regimens, e.g., BCVPP (Carmustine, Cyclophosphamide, Vinblastine, Procarbazine, and Prednisone) in 16 cases, BEACOPP (Bleomycin, Etoposide, Doxorubicin, Cyclophosphamide, Vincristine, Procarbazine, Prednisone) in 7 cases, and R-CHOP (Rituximab, Cyclophosphamide, Doxorubin, Vincristine, Prednisone) in 2 cases.

### 2.1. [^18^F]FDG-PET/CT Protocol and Evaluation

Baseline [^18^F]FDG PET/CT was performed before any therapy, interim PET/CT (iPET/CT) was performed after two cycles of chemotherapy, and end-of-treatment PET/CT (eotPET/CT) after the completion of primary therapy (six cycles of chemotherapy). [^18^F]FDG PET/CT studies were executed following European Association of Nuclear Medicine (EANM) guidelines [14]. [^18^F]FDG PET/CT studies were acquired after at least 6 h of fasting and with glucose blood sample levels less than 150 mg/dl. We injected intravenously an activity of 3.5–4.5 MBq/Kg and the scans were acquired about 60 min after radiotracer injection. Field of acquisition went from mid-thigh to the skull basis. PET scanners used were a Discovery 690 and a ST PET/CT scanner (General Electric Company, GE^®^, Milwaukee, WI, USA). For both scanners, a low-dose free-breathing CT without contrast was acquired for anatomical correlation and attenuation correction. The D-STE acquisition parameters were: 120 kV, fixed tube current ~73 mAs (40–160 mAs), eight slices × 3.75 mm and 3.27 mm interval, pitch 1:5, tube rotation 0.8 s. The D-690 and Biograph True Point acquisition parameters were: 120 kV, fixed tube current ~60 mAs (40–100 mAs), 64 slices × 3.75 mm and 3.27 mm interval, pitch 0.984:1, tube rotation 0.5 s. Baseline PET/CT was executed within 10 days before the first cycle of chemotherapy; interim and end of treatment PET/CT were performed at least 21 days after the last cycle of chemotherapy. The PET images were viewed qualitatively and semi-quantitatively by a nuclear medicine physician with high experience (D.A.). For the visual evaluation of interim and end-of-treatment PET/CT, the Deauville five-point scale was applied. According to these Deauville criteria (DC), [^18^F]FDG PET was reported as follows: score 1 = no uptake present, score 2 = uptake equal to or less than mediastinum, score 3 = uptake more than mediastinum but less than liver, score 4 = uptake moderately high compared to the liver, and score 5 = uptake markedly high compared to the liver. Finally applying DC, [^18^F]FDG PET/CT examinations were defined as a complete metabolic response for scores 1–3 and as an incomplete metabolic response for scores 4–5 [15,16]. Moreover, the same researcher measured MTV and TLG of each baseline PET. MTV was measured staring from the attenuation-corrected [^18^F]FDG PET images using an SUV-based automated contouring program (Advantage Workstation 4.6, GE HealthCare) with an isocounter threshold method based on 41% of the SUVmax, as already recommended by EANM guidelines [14], and total MTV was derived by the sum of all nodal and extranodal lesions with increased uptake. Bone marrow disease was considered in volume measurement only if there was focal uptake; spleen disease was defined if there was focal uptake in the spleen of diffuse splenic uptake higher than 150% of the hepatic background. Subsequently, for the calculation of TLG, we obtained the sum of the product of MTV of each lesion and its SUVmean.

### 2.2. Body Composition Analysis

**Figure 2:**
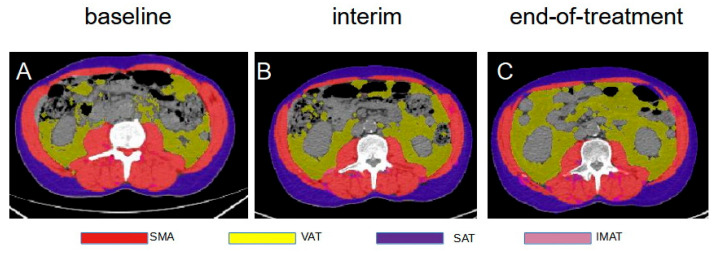
Serial axial CT images of a patient at baseline, interim and end-of-treatment [^18^F]FDG PET/CT with SMA (red), VAT (yellow), SAT (blue) and IMAT (violet) measurements. (**A**) axial image of a baseline scan; (**B**) axial image at the same slice of the interim scan; (**C**) axial image of the same slice of end of treatment scan.

Low-dose CT of [^18^F]FDG PET/CT at baseline, interim, and end-of-treatment was viewed by a physician (F.D.) with previous experience in the measurement of adipose and muscular tissue areas with the free-tool CoreSlicer (www.coreslicer.com) (accessed on 1 January 2022). An axial section at the third lumbar (L3) vertebra with a multiplanar reconstruction was taken for the measurement of the SMA in cm^2^ considering the abdominal transverse rectum, psoas, paraspinal, external and internal obliques muscles and using CT HU cut-offs from –29 to 150. At the same level, subcutaneous, visceral, and intramuscular adipose tissues (SAT, VAT, IMAT) were measured in cm^2^ using HU cut-offs from –190 to –30 for SAT and IMAT, and from –50 to –150 for VAT (Figure 2). Total adipose tissue (TAT) was derived as the total of SAT, IMAT, and VAT. The tissue borders were manually corrected if needed. Then, for each patient, SMA was normalized for the height to derive the skeletal muscle index (SMI) (cm^2^/m^2^). For the definition of sarcopenia, we applied gender-specific cut-offs of SMI of 39 cm^2^/m^2^ for females and 55 cm^2^/m^2^ for male patients, as suggested in the literature [6].

### 2.3. Statistical Analysis

The statistical analyses were carried out using MedCalc Software version 17.1 for Windows (Ostend, Belgium) and Statistical Package for Social Science (SPSS) version 23.0 for Windows (IBM, Chicago, IL, USA). The numeric variables were represented as median, mean, standard deviation, minimum and maximum. The categorical variables were represented as simple and relative frequencies.

The change rates of SMI at interim and end-of-treatment were measured as interim-ΔSMI = (SMI at interim—SMI at baseline)/SMI at baseline ∗ 100. End-of-treatment-ΔSMI = (SMI at end-of-treatment—SMI at baseline)/SMI at baseline ∗ 100. The Mann–Whitney U test and chi-square test were used to compare categorical and continuous variables. A *p*-value < 0.05 was taken as significant. For the evaluation of body composition changes at three different times, repeated analysis of variance (ANOVA) was applied. Progression-free survival (PFS) was measured from the date of baseline [^18^F]FDG PET/CT to the date of first disease progression, relapse, death or the date of the last follow-up. Overall survival (OS) was measured from the date of baseline [^18^F]FDG PET/CT to the date of death from any cause or to the date of the last follow-up. The survival functions were estimated and plotted using the Kaplan–Meier methodology, with a 95% confidence interval (95% CI). Comparison by groups was performed by log-rank test, and the effect of covariates was estimated as hazard ratio (HR) by means of Cox proportional hazard (PH) regression, with 95% CI, either in univariable or multivariable analysis. The Youden index from the ROC curve for survival data was used to find the best cut-off value for all variables.

## 3. Results

### 3.1. Patients Features

Finally, 88 patients were included in this study. Patient features are resumed in Table 1. There was a prevalence of females (*n* = 47). The mean age was 72.8 years (range: 65–91). About half of our patients (*n* = 43) were overweight, followed by normal body mass index (BMI) (*n* = 41) and only 4 cases were underweight. The most frequent HL histological subtype was nodular sclerosis with 46 (52%) patients, followed by mixed cellularity in 23 (26%) patients, lymphocyte rich in five (6%), lymphocyte depletion in four (5%), and not specified in 10 (11%) cases. Patients were staged according to the Ann Arbor system and were categorized in early stages (stage I and II) in 22 cases, and advanced stage (stage III and IV) in the remaining 66. Only seven patients in our study group had bulky disease. Considering the comprehensive geriatric assessment (GCA), 68% were classified as FIT, 23% as UNFIT, and 9% as FRAIL. All baseline PET/CT showed [^18^F]FDG-avid disease with a mean MTV of 129 cm^3^ (4–981 cm^3^) and mean TLG of 1608 (9–11,627). Based on the Lugano classification metabolic response categories, at interim [^18^F]FDG PET/CT 62 (70%) patients had a complete metabolic response (Deauville score 1 in 38 cases, score 2 in 17 cases, and score 3 in seven cases), while the remaining 26 recorded an incomplete metabolic response (Deauville score 4 in 18 and 5 in eight). Instead, after the completion of first-line therapy, at end-of-treatment [^18^F]FDG PET/CT, 67 (76%) patients had complete metabolic response (Deauville score 1 in 56 cases, score 2 in five cases, and score 3 in six cases) and 21 (24%) had incomplete metabolic responses (Deauville score 4 in 4 and 5 in 17).

Among patients with a complete metabolic response at iPET, only eight out of 62 (13%) had an incomplete response at eotPET. On the other hand, among those with an incomplete metabolic response at iPET, 13 out of 26 (50%) passed to a complete metabolic response at eotPET.

### 3.2. Body Composition Changes

**Figure 3:**
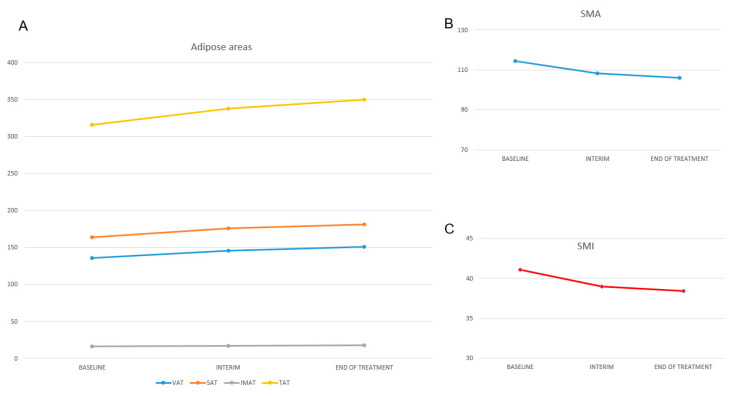
Mean changes in body composition from baseline to interim and end-of-treatment point. (**A**) Average changes in adipose tissues; (**B**) average changes in skeletal muscle area (SMA); (**C**) average changes in skeletal muscle index (SMI).

At baseline, 58 (66%) patients were defined as sarcopenic. The average SMA, SMI, VAT, SAT, IMAT, and TAT were 114.5 cm^2^ (66.8–188.8), 41.4 cm^2^ (22.5–63), 135.5 cm^2^ (6.4–375), 163.7 cm^2^ (4.6–1201), 16.3 cm^2^ (1.2–95), and 315.5 cm^2^ (24–1313), respectively (Table 2). Muscular areas (SMA and SMI) changed significantly during the treatment (*p* < 0.001) (Figure 3). At iPET/CT, the number of sarcopenic patients increased to 68 (77%), while at eotPET/CT increased to 73 (83%). SMA decreased from baseline by 5% at iPET/CT (108.3 cm^2^ vs. 114.5 cm^2^, a reduction of 6.2 cm^2^) and by 7% at eotPET/CT (106 cm^2^ vs. 114.5 cm^2^, a reduction of 8.5 cm^2^). Moreover, SMI decreased by 5% at iPET/CT (39.1 cm^2^/m^2^ vs. 41.4 cm^2^/m^2^, a reduction of 2.3 cm^2^/m^2^) and by 7% at eotPET/CT (41.4 cm^2^/m^2^ vs. 38.4 cm^2/^m^2^, a reduction of 3 cm^2^/m^2^) (Table 2). There were also changes in VAT, SAT, IMAT, and TAT areas (*p* < 0.001) over time (Figure 3). SAT areas increased from baseline at interim by 7% (163.7 cm^2^ vs. 175.6 cm^2^, an increase of 11.9 cm^2^) and by 11% at end of treatment (163.7 cm^2^ vs. 181.3 cm^2^, an increase of 17.6 cm^2^). VAT areas rose at interim by 7% (135.5 cm^2^ vs. 145.2 cm^2^, a gain of 9.7 cm^2^) and by 11% at end of treatment (135.5 cm^2^ vs. 151 cm^2^, a gain of 15.5 cm^2^). IMAT areas rose at interim by 5% (16.3 cm^2^ vs. 17.1 cm^2^, a gain of 0.8 cm^2^) and by 10% at end of treatment (16.3 cm^2^ vs. 18 cm^2^, a gain of 1.7 cm^2^). TAT rose at interim by 7% (315.5 cm^2^ vs. 337.7 cm^2^, a gain of 22.2 cm^2^) and by 11% at end of treatment (315.5 cm^2^ vs. 350 cm^2^, a gain of 34.5 cm^2^).

Baseline sarcopenia was significantly associated with BMI, presence of B symptoms, and CGA status. No significant correlations with the other features were revealed (Table 3). Regarding interim sarcopenia, only BMI and CGA status were confirmed to be significantly correlated (Table 3). Concerning end-of-treatment, only female gender and CGA status were significantly associated with sarcopenia (Table 3).

### 3.3. Prognostic Impact

**Figure 4:**
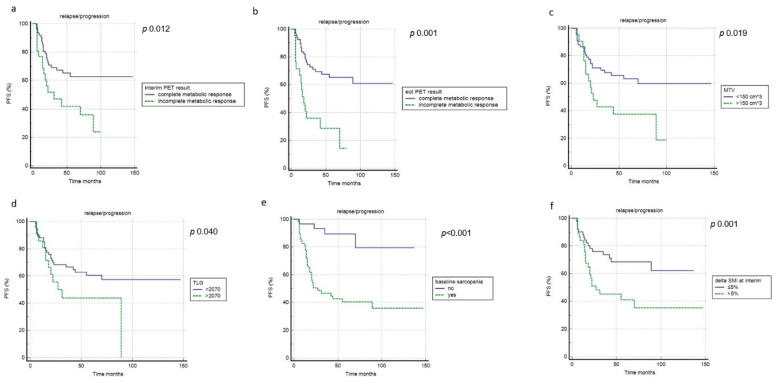
Progression-free survival curves according to interim PET results (**a**), end of treatment PET results (**b**), MTV (**c**), TLG (**d**), baseline sarcopenia (**e**) and ΔSMI at interim (**f**).

**Figure 5:**
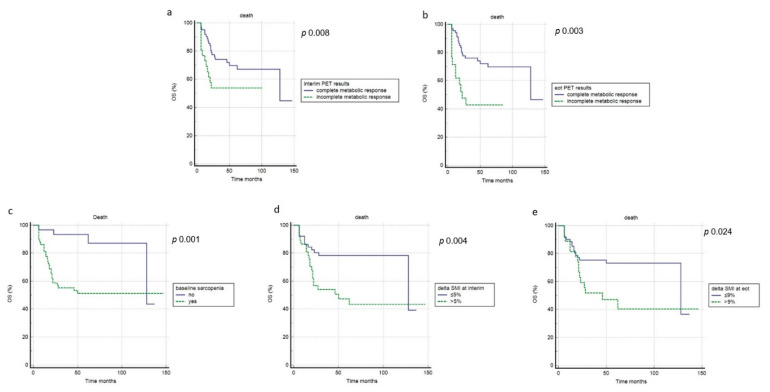
Overall survival curves according to interim PET results (**a**), end of treatment PET results (**b**), baseline sarcopenia (**c**), ΔSMI at interim (**d**), ΔSMI at end of treatment (**e**).

At a median follow-up of 47.5 months (range 6–147 months), 38 patients (43%) registered relapse or progression of disease), with a mean time of 21 months (range: 5–89 months), while death happened in 32 cases (36%), with an average time of 22 months (range: 6–128). Overall, the one- and three-year PFS rates were 86% and 57%. However, the one- and three-year OS rates were 85% and 66%. Regarding PFS, in univariate analysis, the interim and end-of-treatment metabolic response, MTV, TLG, age, presence of B symptoms, baseline sarcopenia, and interim-ΔSMI were significantly associated with outcome, while the other features were not (Figure 4, Table 4). In multivariate analysis, interim and end-of-treatment metabolic response, MTV, TLG, and baseline sarcopenia were confirmed to be independent prognostic factors. Concerning OS, chemotherapy protocol, age, the metabolic response at interim and end-of-treatment PET, baseline sarcopenia, interim-ΔSMI, and end-of-treatment-ΔSMI were significantly related to the outcome at univariate analysis, but only metabolic response at interim, end-of-treatment PET, baseline sarcopenia, interim-ΔSMI, and end-of-treatment-ΔSMI were confirmed at multivariate analysis, while SMI showed no prognostic impact (Figure 5, Table 4).

## 4. Discussion

Sarcopenia is usually defined as a muscle atrophy with a decrease in muscular cell number and size and it is mainly caused by the presence of a systemic inflammatory response that brings protein degradation. The pathology of HL consists of malignant cells of Reed–Sternberg cells enclosed by a larger pool of inflammatory cells which include nonmalignant lymphocytes, neutrophils macrophages, eosinophils, and plasma cells [17,18,19]. This chronic inflammation can be associated with the loss of muscle mass and strength. Moreover, age and treatment may also stimulate muscle wasting, especially in elderly patients. The open question concerns whether these body changes have an impact on patient survival or not. In our manuscript, we choose to investigate elderly HL patients, because they are normally underestimated in clinical studies on HL. However, this presents a real absolute clinical challenge, due to the rising incidence of HL in this range of age that is expected in the next decades due to the general ageing of Western populations, as well as the difficulty of balancing the risk of toxicities and the goal of efficacy against this background. Moreover, elderly HLs have peculiar features and present a difficult balance between physiological muscle deterioration and pathological conditions which may cause severe sarcopenia. For these reasons, we prefer to choose the selected population for this kind of analysis. Very few studies have been published so far about the prognostic role of sarcopenia in elderly lymphoma and in most cases were based upon DLBCL [6]. Specific studies about elderly HL are scarce [20]. Zilioli et al. [20] found a positive correlation of sarcopenia measured as a static parameter with PFS and OS only in men. However, the role of body composition changes during treatment is not yet clear. In our analysis, the rate of sarcopenia patients increased significantly during the course of treatment, passing from 66% at baseline (before chemotherapy) to 77% at interim after two cycles of chemotherapy and 83% at the end of the treatment (six cycles of chemotherapy). This is directly related to the reduction of SMI. The major reduction happened early after two cycles of chemotherapy, meaning a rapid muscle decline after chemotherapy. This diminution continues but less strongly until the end of first-line treatment. The reduction of SMA and SMI and consequent sarcopenia gain during the course of treatment was already demonstrated by other studies [10,11,12,13]. Compared to these studies where the rate of sarcopenia at baseline ranged from 27% to 55%, the rate of sarcopenia in our population was higher with 66% at baseline. This is due to the advanced age (>65 years) of our patients. The only paper specific to older lymphoma [12] reported a rate of sarcopenia of 55%, but they included many lymphoma variants (only eight HL) and the age criterion of inclusion was superior to 50 years, lower than us. For these reasons, a direct comparison with other studies seems to be difficult and excessive. Baseline sarcopenia was significantly associated both with PFS and OS at multivariate analysis. Besides sarcopenia, the muscular changes during treatment expressed as ΔSMI (both at interim and end-of-treatment) were also demonstrated to be independently correlated with OS. Previously, no other studies evaluated this point. A higher reduction of SMI after chemotherapy may cause a weakening in the patient, with a reduction of tolerance to therapy and a high risk of side effects/toxicities potentially fatal. Beyond muscle areas (SMA and SMI) which are fundamental for the definition of sarcopenia, we measured also adipose tissue expressed as SAT, TAT, IMAT, and TAT, observing an increase in these areas during the course of treatment. SAT and VAT were the parameters that increased more (5% at interim and 11% at end of treatment). This is a direct consequence of the reduction of SMA, with more “space” for adipose tissue. Moreover, the treatment response of patients with abdominal disease also decreased or disappeared, which may explain this gain of adipose areas. The potential clinical meaning of adipose tissue areas remains unclear. Only Jabbour et al. [10] demonstrated that sarcopenic obesity, defined as VAT/SMI ratio, present three months after hematopoietic stem cell transplantation, was a predictor of death. Bas et al. [21] made the first attempt to estimate body changes during treatment using some metabolic features such as MTV. They demonstrated a faint reduction of mean MTV (from 4.13 mm3 to 4.10 mm3) after therapy, and this decline was associated with advanced age and elevated BMI. For elderly patients, a simple geriatric scale based on the patient’s fitness status could be helpful to define patients’ risks to toxicities and the efficacy of treatments. For this reason, a simplified comprehensive geriatric assessment (CGA) was introduced by the Fondazione Italiana Linfomi (FIL), based on a model that categorizes the patients into three classes (FIT, UNFIT, and FRAIL) according to activities of daily living (ADL), age instrumental ADL, and the Cumulative Illness Rating Scale for Geriatrics. This model was previously validated in NHL [22,23,24], and not in HL. In our analysis, we derived a direct relation between sarcopenia and CGA status. The rate of UNFIT-FRAIL patients was significantly larger in patients with sarcopenia at different times than in patients without sarcopenia. This could be due to the definition of CGA, which consists of age and performance status abilities. Instead, sarcopenia was not influenced by chemotherapy protocol. Aside from sarcopenia, [18F]FDG PET/CT features also showed a strong power in the evaluation of prognosis in elderly HL. The Deauville criteria and derived Lugano recommendations [15,16] demonstrated prognostic impact in several lymphomas and are routinely applied in HL, FL, and DLBCL [25]. Recent evidence also underlined a potential positive impact in other lymphoma variants, e.g., Burkitt lymphoma [26], mantle cell lymphoma [27], and central nervous system lymphoma [28]. Moreover, in the population of this study (elderly HL), Deauville criteria were confirmed to be significantly correlated with PFS and OS, both at interim and end-of-treatment. These results established again the need to adopt a PET/CT-driven therapeutic approach, with the possibility to personalize the management of patients according to PET findings. PET/CT could be useful also in guiding the therapeutic strategy, with the strength to avoid unneeded and toxic therapies, to identify early the need for second-line therapeutic protocols, considering that elderly patients may be ineligible for certain aggressive high dose chemotherapy and autograft treatments. Elderly patients with a positive iPET or eotPET should be considered for further treatment or initiation of different treatment plans. PET findings may cooperate with the classical clinical prognostic factors for predicting survival. Regarding semiquantitative parameters, MTC and TLG have emerged as prognostic factors in lymphoproliferative diseases [29]. In out manuscript, MTV and TLG predicted PFS, but not OS. This is probably because, in elderly people, other factors, e.g., the same age or comorbidities, may affect survival. Probably, the idea to apply MTV and TLG in the clinical practice is excessive and premature, mainly due to the lack of a standardized methodology for their evaluation. Nowadays, several methods are proposed to make these measurements, with different threshold methods, e.g., a fixed absolute threshold or fixed relative threshold, or adaptive threshold, or background threshold taking blood pool or liver as reference. Besides, until now, there has been no shared consensus regarding the technical references to adopt. Here, we choose the method suggested by EANM guidelines [14], but this is strongly debated. Some limitations reduce the quality of this manuscript, such as the retrospective nature of the study, the heterogeneous management received by our patients (such as primary therapy), and the relatively low sample of patients recruited directly related to the inclusion criteria applied. Prospective studies are warranted to controvert or confirm our results in real-life settings.

## 5. Conclusions

The incidence of sarcopenia was found to be increased in elderly HL during chemotherapy course, with, e.g., the gain of adipose tissue areas. [^18^F]FDG PET/CT features (metabolic response at interim and end-of-treatment), baseline sarcopenia, and body composition changes during chemotherapy may have a prognostic role.

## Figures and Tables

**Table 1:** Patients’ characteristics.

Characteristics	Mean ± SD (Range)	*n* (%)
Age, years	72.8 ± 5.6 (65–91)	
Gender		
Female		47 (53%)
Male		41 (47%)
BMI kg/m^2^	24.9 ± 4.7 (16.9–41.1)	
Normal		41 (47%)
Overweight		43 (49%)
Underweight		4 (5%)
Symptoms B		40 (45%)
Bulky disease		7 (7%)
LDH > normal level		50 (57%)
Stage		
I-II		22 (25%)
III-IV		66 (75%)
Histology		
Nodular sclerosis		46 (52%)
Mixed cellularity		23 (26%)
Lymphocyte rich		5 (6%)
Lymphocyte deplete		4 (5%)
Not specified		10 (11%)
CGA		
FIT		60 (68%)
UNFIT		20 (23%)
FRAIL		8 (9%)
Treatment		
ABVD/ABVD-like		63 (72%)
BCVPP		16 (18%)
BEACOPP		7 (7%)
R-CHOP		2 (3%)
Interim PET/CT results		
DS 1–3		62 (70%)
DS 4–5		26 (30%)
End-of-treatment PET/CT results		
DS 1–3		67 (76%)
DS 4–5		21 (24%)
Baseline MTV	129.9 ± 150.6 (4–981)	
Baseline TLG	1608 ± 1859 (9–11,627)	

BMI: body mass index; LDH = lactate dehydrogenase; CGA: comprehensive geriatric assessment; ABVD: Doxorubicin, Bleomycin, Vinblastine, Dacarbazine; BCVPP: Carmustine, Cyclophosphamide, Vinblastine, Procarbazine, and Prednisone; BEACOPP: Bleomycin, Etoposide, Doxorubicin, Cyclophosphamide, Vincristine, Procarbazine, Prednisone; R-CHOP: Rituximab, Cyclophosphamide, Doxorubin, Vincristine, Prednisone; DS: Deauville score; MTV: metabolic tumor volume; TLG: total lesion glycolysis; SD = standard deviation.

**Table 2:** Body composition features at different times.

Variable	Baseline	Interim	End-of-Treatment
SMA (cm^2^), mean ± SD (range)	114.5 ± 28.8 (66.8–188.8)	108.3 ± 26.2 (66–172)	106 ± 26 (62.6–171)
Δ% from baseline		−5%	−7%
SMI (cm^2^/m^2^), mean ± SD (range)	41.4 ± 8.9 (22.5–63)	39.1 ± 8.1 (22.3–57)	38.4 ± 8.3 (21–56.7)
Δ% from baseline		−5%	−7%
VAT (cm^2^), mean ± SD (range)	135.5 ± 89.1 (6.4–375)	145.2 ± 93.6 (7.9–385)	151 ± 96.3 (8.9–399)
Δ% from baseline		+7%	+11%
SAT (cm^2^), mean ± SD (range)	163.7 ± 136 (14.6–1201)	175.6 ± 150.8 (16.5–1350)	181.3 ± 153.3 (18–1360)
Δ% from baseline		+7%	+11%
IMAT (cm^2^), mean ± SD (range)	16.3 ± 13.5 (1.2–95)	17.1 ± 13.8 (1.35–98)	18 ± 14 (1.5–98)
Δ% from baseline		+5%	+10%
TAT (cm^2^), mean ± SD (range)	315.5 ± 182.9 (24–1313)	337.7 ± 198 (24–1468)	350 ± 202.5 (24–1485)
Δ% from baseline		+7%	+11%
Sarcopenia, *n* (%)	58 (66%)	68 (77%)	73 (83%)
Δ% from baseline		+11%	+17%

SMA: skeletal muscle area; SMI: skeletal muscle index; VAT: visceral adipose tissue; SAT: subcutaneous adipose tissue; IMAT: intramuscular adipose tissue; TAT: total adipose tissue.

**Table 3:** Comparison between sarcopenic and not sarcopenic patients at baseline.

Variable	Baseline		
	Sarcopenia *n* = 58	Not Sarcopenia *n* = 30	*p* Value
Age (average ± SD)	72.6 ± 5.7	73.2 ± 5	0.697
Female:male	29:29	18:12	0.258
BMI (average ± SD)	23.8 ± 3.7	27.3 ± 5.5	**<0.001**
Tumor stage III-IV	43 (74%)	23 (77%)	0.518
Symptoms B	32 (55%)	8 (27%)	**0.011**
Bulky disease	6 (10%)	1 (3%)	0.278
LDH > normal level	28 (47%)	12 (40%)	0.832
Nodular sclerosis variant	29 (50%)	17 (57%)	0.091
CGA FIT:UNFIT:FRAIL	37:14:7	23:6:1	**0.019**
Treatment ABVD/ABVD like	18 (31%)	8 (27%)	0.674
iPET complete metabolic response	41 (71%)	21 (70%)	0.443
eotPET complete metabolic response	42 (72%)	25 (83%)	0.312
Baseline MTV (average ± SD)	139.4 ± 137.7	110.7 ± 161.9	0.404
Baseline TLG (average ± SD)	1682 ± 1729	1459 ± 1916	0.600
	**Interim**		
	Sarcopenia *n* = 68	Not sarcopenia *n* = 20	
Age (average ± SD)	72.8 ± 5.7	72.85 ± 5.3	0.993
Female:male	33:35	14:6	0.092
BMI (average ± SD)	24.1 ± 4	27.8 ± 5.8	**0.001**
Tumor stage III-IV	49 (73%)	17 (85%)	0.562
Symptoms B	33 (48%)	7 (35%)	0.298
Bulky disease	6 (9%)	1 (5%)	0.583
LDH > normal level	32 (47%)	8 (40%)	0.767
Nodular sclerosis variant	34 (50%)	12 (60%)	0.316
CGA FIT:UNFIT:FRAIL	43:18:7	17:2:1	**0.009**
Treatment ABVD/ABVD like	21 (31%)	5 (25%)	0.617
eotPET complete metabolic response	52 (76%)	15 (75%)	0.893
Baseline MTV (average ± SD)	131.7 ± 130	123.9 ± 208.8	0.840
Baseline TLG (average ± SD)	1627 ± 637	1545 ± 2522	0.863
	**End of treatment**		
	Sarcopenia *n* = 73	Not sarcopenia *n* = 15	
Age (average ± SD)	72.5 ± 5.6	74.3 ± 5.3	0.256
Female:male	35:38	12:3	**0.023**
BMI (average ± SD)	24.6 ± 4.4	27 ± 5.8	0.065
Tumor stage III-IV	56 (77%)	10 (67%)	0.871
Symptoms B	35 (48%)	5 (33%)	0.645
Bulky disease	7 (14%)	0 (0%)	0.215
LDH > normal level	36 (49%)	4 (27%)	0.798
Nodular sclerosis variant	38 (52%)	8 (53%)	0.248
CGA FIT:UNFIT:FRAIL	47:18:8	13:2:0	**0.003**
Treatment ABVD/ABVD like	21 (29%)	5 (33%)	0.727
Baseline MTV (average ± SD)	142.2 ± 161	69.9 ± 47.4	0.090
Baseline TLG (average ± SD)	1751 ± 1988	912 ± 725	0.111

BMI: body mass index; LDH = lactate dehydrogenase; CGA: comprehensive geriatric assessment; ABVD: Doxorubicin, Bleomycin, Vinblastine, Dacarbazine; MTV: metabolic tumor volume; TLG: total lesion glycolysis; SD = standard deviation.

**Table 4:** Univariate and multivariate analyses for PFS and OS.

Variable	Univariate Analysis		Multivariate Analysis	
	HR (95% CI)	*p* Value	HR (95% CI)	*p* Value
**PFS**				
Gender	1.528 (0.800–2.925)	0.198		
Age > 75	2.021 (1.026–3.979)	**0.041**	1.314 (0.667–2.590)	0.429
BMI	1.989 (0.450–3.456)	0.450		
Tumor Stage	1.704 (0.837–3.471)	0.145		
Symptoms B	1.970 (1.026–3.783)	**0.041**	1.807 (0.931–3.508)	0.080
LDH level > normal value	1.340 (0.701–2.345)	0.222		
Bulky disease	1.546 (0.457–5.221)	0.484		
Nodular sclerosis variant	0.995 (0.520–1.899)	0.989		
CGA FIT	0.785 (0.393–1.570)	0.495		
Treatment ABVD/ABVD like	1.918 (0.911–4.038)	0.086		
iPET complete metabolic response	1.259 (1.230–5.464)	**0.012**	2.878 (1.340–5.009)	**0.023**
eotPET complete metabolic response	5.888 (2.448–14.181)	**0.001**	3.303 (1.571–6.940)	**0.001**
MTV > 150 cm^3^	2.547 (1.163–5.579)	**0.019**	2.085 (1.062–4.092)	**0.032**
TLG > 2070	2.936 (1.876–4.278)	**0.040**	2.430 (1.201–4.332)	**0.043**
Baseline sarcopenia	3.725 (1.934–7.174)	**<0.001**	3.881 (1.009–7.021)	**0.001**
Interim-ΔSMI > 5%	2.391 (1.230–4.646)	**0.010**	3.768 (0.800–5-987)	0.124
End-of-treatment-ΔSMI > 9%	1.909 (0.923–3.950)	0.081		
**OS**				
Gender	1.098 (9.537–2.246)	0.796		
Age > 75	2.929 (1.381–6.213)	**0.005**	1.545 (0.682–3.492)	0.297
BMI	1.888 (0.560–2.987)	0.230		
Tumor Stage	2.007 (0.918–4.383)	0.080		
Symptoms B	1.660 (0.807–3.412)	0.168		
LDH level > normal value	0.685 (0.386–1.909)	0.546		
Bulky disease	1.546 (0.457–5.221)	0.484		
Nodular sclerosis variant	0.884 (0.437–1.787)	0.732		
CGA FIT	1.520 (0.711–3.250)	0.279		
Treatment ABVD/ABVD like	4.473 (1.949–10.263)	**<0.001**	2.874 (0.309–6.310)	0.222
iPET complete metabolic response	2.067 (1.090–4.669)	**0.008**	2.222 (1.101–5.234)	**0.012**
eotPET complete metabolic response	3.890 (1.554–9.740)	**0.003**	2.402 (1.080–5.336)	**0.019**
MTV > 150 cm^3^	1.346 (0.582–3.109)	0.483		
TLG > 2070	2.118 (0.904–4.966)	0.084		
Baseline sarcopenia	3.275 (1.592–6.737)	**0.001**	3.661 (1.111–7.321)	**0.001**
Interim-ΔSMI > 5%	2.789 (1.365–5.737)	**0.004**	2.324 (1.130–4.021)	**0.009**
End-of-treatment-ΔSMI >9%	2.438 (1.124–5.288)	**0.024**	4.001 (2.245–6.091)	**0.031**

PFS: progression-free survival; OS: overall survival; HR: hazard ratio; CI: confidence interval; BMI: body mass index; CGA: comprehensive geriatric assessment; ABVD: Doxorubicin, Bleomycin, Vinblastine, Dacarbazine; iPET: interim PET; eotPET: end of treatment PET; MTV: total metabolic tumor volume; TLG: total lesion glycolysis; SMI: skeletal muscle index.

## Data Availability

The data can be shared up on request.
